# Knowledge, attitudes and practices of Moroccan cancer patients and their relatives towards the COVID-19 pandemic

**DOI:** 10.4314/ahs.v23i4.14

**Published:** 2023-12

**Authors:** Nadia Nouari, Saloua Lamtali, Majda Sebbani, Mouna Khouchani, Mohamed Amine, Latifa Adarmouch

**Affiliations:** 1 Community Medicine and Public Health Department, Biosciences and Health science Laboratory, Faculty of Medicine and Pharmacy, Cadi Ayyad University, Marrakesh, Morroco; 2 Higher Institute of Nursing and Health techniques, Marrakesh, Morocco (ISPITS-M); 3 Laboratory of Pharmacology, Neurobiology, Anthropobiology and Environment (LPNAE) Faculty of Sciences Semlalia, University Cadi Ayyad, Marrakesh Morocco; 4 Clinical Research department, Mohammed VI University Hospital, Marrakesh Morocco; 5 Morpho sciences research laboratory, Faculty of Medicine and Pharmacy, Cadi Ayyad University

**Keywords:** Perceptions, knowledge, attitudes, practice, cancer, coronavirus, SARS-CoV-2, pandemic, infectious disease, COVID-19, public health

## Abstract

**Background:**

This study aims to describe the knowledge, attitudes, and practices of cancer patients and their relatives regarding the COVID-19 pandemic in Morocco.

**Methods:**

A self-administered online questionnaire was administrated to 133 adults aged 19 to 70 years old, including 32 cancer patients and 101 people from the relatives of cancer patients.

**Results:**

The participants showed a very good level of knowledge (cancer patients (84.6%), relatives (82.7%)) and practice (cancer patients (96.0%), relatives (85%)) regarding covid-19. . A very large proportion of cancer patients (84.4%) were very worried about the virus, compared with only 52.5% of relatives. They were concerned about the potential risk of infection and felt personally exposed (93.8%) to serious complications from COVID-19. The knowledge, attitudes and practices score were significantly associated with age (p=0,018), sex (p=0.002), professional activity (p=0,036), medical insurance (p=0,009), place of residence (p= 0,017), presence or absence of cancer (p=0,000), and perception of the danger of catching COVID-19 (p=0,041),

**Conclusions:**

Although the level of knowledge and practices of cancer patients and relatives was very satisfying, disparities between the two groups were still to be noted. Cancer patients go out less and practice more, despite the impact of confinement on their health

## Introduction

A new variant of coronavirus under the name of severe acute respiratory syndrome-coronavirus-2 (SARS-CoV-2) was identified for the first time in humans in the city of Wuhan, China. The new infection was declared by the WHO as a pandemic on March 11, 2020 1,2 In just a few months, the pandemic struck almost the entire globe. As a result, the number of infections and deaths caused by COVID-19 has steadily increased in the 200 countries and territories impacted by the pandemic 3.

In Morocco, the total number of SARS-CoV-2 contaminations rose to 502,102 confirmed cases and 8,900 deaths by April 11, 2020 4. Following the experiences of other countries, Morocco realized very early on the importance of having a strategy to control the epidemiological curve as a way of stemming the spread of the pandemic. Especially the state of health emergency was announced on March 19, 2020 and a series of drastic measures were implemented, including containment measures, social distancing, individual protection and prevention measures^5^,^6^. A communication plan was deployed to accompany the interventions implemented as part of public health response to the pandemic 6. Its purpose was to push citizens to respect these measures even within their homes. Respecting these measures, is very important, particularly among at-risk and most vulnerable groups 7.

Individuals with underlying health problems are at higher risk of serious complications and death related to COVID-19. The vulnerability to infectious diseases is also associated with age, race, ethnicity, socioeconomic status and difficulties in accessing clear information about their health. According to studies, the risk of having SARS COV-2 infection is much higher among cancer patients or those with a history of cancer, than among the general population, due to their immunosuppressed status or the disease itself. Moreover, this status exposes them to severe forms of complications from COVID-19, to 3.5 times the risk of requiring mechanical ventilation or admission to intensive care units, and to the risk of death. Patients who had received chemotherapy or undergone surgery within 30 days before being infected by COVID-19 had a higher risk of serious events than patients who had not been treated with chemotherapy or had not undergone surgery. In addition to the negative impact on the prognosis of cancer disease, as a result of unnecessary hospitalizations, the delays in treatment and difficulties to have access to cancer care, in this pandemic context. 8-12.

On the other hand, timely, reliable, consistent, and useful information will surely help the public to make conscious decisions and promote their well-being 13,14. The lack of sufficient information about the virus, about its mode of transmission and about on individual protection measures, has proven to be a factor favouring the contamination and the spread of the pandemic among cancer patients and caregivers 15. Access to information on public health issues allows people to promote their health, help them to understand the disease and treatment and to better cooperate with caregivers. It enables them to be resources contributing to improving their health 14.

At this time, there is no specific treatment or universal vaccine against all the new variants of SARS-CoV-2. in fact, despite the existence of a vaccine against covid19, researchers estimate that at least 80% of the population should be vaccinated in order to achieve herd immunity, something that is difficult to achieve quickly during the course of this year 2021^16^,^17^. the evolution of the pandemic therefore depends essentially on the behaviour of the population. This behavior is influenced by people's knowledge, attitudes and practices towards the disease, their level of emotion and panic, their perceptions and the degree of application of safety and personal protection measures 18-21.

This study aimed first to determine the knowledge, the attitudes and the practices (KAPs) of cancer patients and cancer patients' relatives towards the COVID-19 pandemic, then, to identify the factors associated with these elements, and the possible differences between these two groups.

In view of the rarity of studies on the same subject, this survey requires great importance. Its results would contribute to the Moroccan government's efforts to better counteract the COVID-19 pandemic among cancer patients and at-risk groups.

## Methods

This cross-sectional on-line survey was conducted in Morocco between May, 20 2020 and June, 5 2020. It was impossible to conduct a face-to-face survey because of the containment measures during the COVID-19 pandemic. Cancer patients or relatives of cancer patients were targeted by this survey. The questionnaire was intended for all Moroccan volunteers regardless of their location. Recruitment was performed via the network of health professionals, thematic associations and patient associations. An invitation was published online via social media (WhatsApp, Facebook…).

The questionnaire included data on several fields. Socio-demographic data included age, gender, marital status, educational level, professional activity, residency area (rural/urban), medical insurance and monthly income. Data on the health status of participants included questions for cancer patients on whether they had been diagnosed with cancer or a chronic disease, the stage of cancer treatment, the type of cancer, difficulties with follow-up, check-ups and continuity of cancer care, and other questions for relatives on whether or not they had a chronic disease, the presence of a cancer patient in the family and the relationship with the cancer patient.

A second part of the questionnaire included questions on knowledge about the COVID-19 (infectious risk, sources of information, symptoms, mode of transmission, preventive measures). The third part of the questionnaire concerned the attitudes adopted during confinement towards the measures taken by the state, towards unruly people and the degree of impact of confinement on the health of the participants. The fourth and last part was reserved for the degree of application of safety and personal protection measures by participants during containment, the changes observed in their habits and relationships with their entourage, access to care for cancer patients and compliance with diet prescribed by the doctor.

The survey data were collected using an online questionnaire that was structured and developed by the research team. An online survey portal, Google Form, was used and participants were invited to complete and submit the form. All participants were invited to express their consent to participate. The recruitment invitation contained a brief introduction on the context, purpose, voluntariness of participation, declarations of anonymity and confidentiality, instructions for completing the questionnaire, and the link to the online questionnaire. The questionnaire was designed in Arabic, the native language of Moroccans. It was initially tested among a group of participants who were later excluded from the study.

Knowledge about COVID-19 was assessed using 26 multiple-choice questions. A total cumulative knowledge score was calculated for each participant. Questions were awarded one point for the correct answer and zero for the unanswered or incorrect answers. The minimum score was 0 and the maximum was 26. A score more or equal to 13 points, indicated greater knowledge of COVID-19. The application of safety and personal protective measures during containment were assessed using 16 multiple-choice questions. A total cumulative of good practices score was calculated for each participant. Questions with a correct answer received three points for the answer “always”, two points for the answer “frequently”, one point for the answer “rarely” and zero for the answer “never”, and conversely for the incorrect answers (three points for the answer “never”, two points for the answer “rarely”, one point for the answer “frequently” and zero for the answer “always”). The minimum score was 0 and the maximum was 48. A score greater than or equal to 24 indicated better application of COVID-19 preventive measures.

Statistical data analyses were conducted using IBM Statistical Package for the Social Sciences (SPSS) version 16.0. Thus, the analysed sample consisted of 133 Moroccan participants.

Data were described using means and standard deviation for quantitative variables and frequencies for categorical one. We performed uni-variate and multivariate analyses to identify factors associated with good KAPs. As a result, Mann-Whitney test was used to determine correlations between, on the one hand, knowledges and practice's scores, number of outings outside the house per week, impact of containment on participants' health and, on the other hand, socio-demographic variables and perception of danger related to COVID-19. The comparison of scores between the cancer patients and the relatives' groups was based on analysis of the chi-square test of variance. The level of statistical significance was set at 0.05. Finally, the analysed data were organized and presented in tabular and narrative form.

## Results

A total of 133 participants completed the survey. 32 (24.06%) of them were cancer patients (sick) and 101 (75.93%) are relatives of cancer patients (not sick). Indeed, 51% of these relatives have at least one very close relative who is a cancer patient, whether it is a parent, a child or siblings (brother or sister). 15(47%) of cancer patients suffer from breast cancer. Among the cancer patients 23(72%) have already completed their cancer treatment protocol, while 14 (44%) have at least one additional chronic disease. The average age was 46.7±12.2 years for cancer patients and 36.7±11.4 years for relatives with a minimum of 19 years and a maximum of 70 years for all participants. Other demographic characteristics are presented in Table 01.

**Table 01:** Socio-demographic characteristics of Moroccan cancer patients and the relatives between May, 20 2020 and June, 5 2020

Variables		Cancer patients group N (%) ^a^	Relatives group N (%)
Distribution of participants		32 (24.1)	101 (75.9)
Sex	Male	08 (25.0)	33 (32.7)
	Female	24 (75.0)	68 (67.3)
Marital status	Single	02 (6.3)	45 (44.6)
	Others	30 (93.7)	56 (55.4)
Level of education	Academic	09 (28.1)	93 (92.1)
	Others	23 (71.9)	08 (7.9)
Environment	Urban	29 (90.6)	89 (88.1)
	Rural	03 (9.4)	12 (11.9)
City	Region Marrakesh Safi	22 (68.8)	59 (58.4)
	Other regions	10 (31.2)	42 (41.6)
Medical insurance	Yes	28 (87.5)	86 (85.1)
	No	04 (12.5)	15 (14.9)
Professional activity	Active	16 (50)	78 (77.2)
	Not active	16 (50)	23 (22.8)
Total household income (per month)	≤3000 dh^b^	09 (28.1)	12 (12)
	>3000 dh	23 (71.9)	88 (88)^c^

a frequency, %: Percentage

b dirhams (01dh-0.11 dollar American)

c 1 (missing)

Source: Prepared by the authors from the study results

According to the results of the study, none of the participants were found to have been infected with the virus up to the survey ‘s date. All participants stated that they had heard about COVID-19 since the beginning of the pandemic in Morocco, through official media for cancer patients or social media for relatives (Supplementary table 01 and Supplementary table 02).

**Supplementary table 01:** Sources of COVID-19 Announcement Information for the 1^st^ Time in Morocco Used by Participants between May, 20 2020 and June, 5 2020:

Sources of information on the COVID-19 announcement	Cancer patients group N (%)	Relatives group N (%)
Health care professionals	08 (25,0)	15 (14.9)
Ministry of Health website	0	2 (02.0)
National TV channels	23 (71,9)	16 (15.8)
International TV channels	0	24 (23.8)
National and regional radio stations	0	0
Social media (Facebook, Instagram, WhatsApp…)	01(03,1)	31 (30.7)
The internet (YouTube, other websites…)	0	12 (11,9)
Newspapers	0	0
Word of mouth: (family, neighbours…)	0	01 (1.0)

Source: Prepared by the authors from the study results

**Supplementary table 02:** Most reliable sources of COVID-19 information for Moroccan cancer patients and the relatives between May, 20 2020 and June, 5 2020

Most reliable sources of information	Cancer patients' group		Relatives group	
	Yes	No	Yes	No
	N (%)	N (%)	N (%)	N (%)
Decision-makers and Ministry of Health officials	29 (90.6)	03 (9.4)	69 (68.3)	32 (31.7)
Health professionals (nurses, doctors…)	23 (71.9)	09 (28.1)	29 (28.7)	72 (71.3)
The website of the Ministry of Health	23 (71.9)	09 (28.1)	47 (46.5)	54 (53.5)
The website of the Ministry of the Interior	19 (59.4)	13 (40.6)	17 (16.8)	84 (83.2)
National TV channels	29 (90.6)	03 (9.4)	40 (39.6)	61 (60.4)
International TV channels	01 (3.1)	31 (96.9)	24 (23.8)	77 (76.2)
National and regional radio stations	21 (65.6)	11 (34.4)	17 (16.8)	84 (83.2)
Facebook	0	32 (100.0)	07 (06.9)	94 (93.1)
WhatsApp	0	32 (100.0)	05 (05.0)	96 (95.0)
Instagram	0	32 (100.0)	02 (02.0)	99 (98.0)
The internet (YouTube, other websites…)	0	32 (100.0)	06 (05.9)	95 (94.1)
Newspapers	01 (3.1)	31 (96.9)	02 (02.0)	99 (98.0)
Word of mouth: (family, neighbours…)	0	32 (100.0)	02 (02.0)	99 (98.0)

Source: Prepared by the authors from the study results

The rates of correct response for the 26 questions on COVID-19 knowledge were ranging from 65.3% to 100% among cancer patients, and 30.8% to 100% among relatives. The mean knowledge score on COVID-19 was 22.0±2.9 for cancer patients and 21.5±3.1 for relatives (0min-26max). In addition, the global rate of response was 84.6% for cancer patients, and 82.7% for relatives (mean score*100/total score). All of the cancer patients and 99% of the relatives were able to obtain scores above 13, which represents a very high level of knowledge about COVID-19.

The knowledge score among cancer patients differed significantly by professional activity, Active cancer patients were more knowledgeable about COVID-19 than non-active cancer patients (p<0.05). The knowledge score among relatives differed according to place of residence and medical insurance (P<0.05). Relatives from urban areas or with medical insurance were more knowledgeable about COVID-19 than those from rural areas or without medical insurance The results of the evaluation of participants' knowledge of symptoms, mode of transmission and individual safety and prevention measures against COVID-19 are presented in Supplementary table 03.

**Supplementary table 03:** Assessment of Moroccan participants' knowledge between May, 20 2020 and June, 5 2020

Knowledge		Cancer patients' group		Relatives group	
		Yes	No	Yes	No
		N (%)	N (%)	N (%)	N (%)
**Symptoms**	-Fever	32(100)	0(0)	95(94.1)	6(5.9)
	- Breathing difficulty	29(90.6)	3(9.4)	97(96)	4(4)
	- Dry cough	29(90.6)	3(9.4)	89(88.1)	12(11.9)
	- Fatigue	18(56.2)	14(43.8)	78(77.2)	23(22.8)
	- Sore throat	24(75)	8(25)	78(77.2)	23(22.8)
	- Dental pain	31(96.9)	1(3.1)	100(99)	1(1)
	- Muscle pain	12(37.5)	20(62.5)	45(44.6)	56(55.4)
	- Nasal discharge	25(78.1)	7(21.9)	19(18.8)	82(81.2)
	- Loss of sense of smell and taste	24(75)	8(25)	69(68.3)	32(31.7)
	- Diarrhea	14(43.8)	18(56.2)	55(54.5)	46(45.5)
**Transmission mode**	- Droplets from the nose or mouth?	32(100)	0(0)	100(99)	1(1)
	- Direct physical contact with sick people?	32(100)	0(0)	92(91.1)	9(8.9)
	- Direct contact with infected objects or surfaces?	32(100)	0(0)	87(86.1)	14(13.9)
	- Direct contact with infected money or papers?	24(75)	8(25)	83(82.2)	18(17.8)
	- Direct contact with an asymptomatic person?	19(59.4)	13(40.6)	73(72.3)	28(27.7)
**Personal protective measures already known by the participants**	- Staying home and going out only when needed	32(100)	0(0)	96(95)	5(5)
	- Frequent hand washing with soap and water	32(100)	0(0)	98(97)	3(3)
	- The use of alcoholic disinfectant for hand disinfection when necessary	31(96.9)	1(3.1)	92(91.1)	9(8.9)
	- Fumigation of the house with medicinal plants (eucalyptus and chih)	25(78.1)	7(21.9)	79(78.2)	22(21.8)
	- Disinfection of surfaces with diluted bleach	27(84.4)	5(15.6)	84(83.2)	17(16.8)
	- Mandatory wearing of masks	29(90.6)	3(9.4)	90(89.1)	11(10.9)
	- Avoid touching your eyes, nose and mouth before washing your hands.	26(81.2)	6(18.8)	90(89.1)	11(10.9)
	- Maintain a safety distance of at least one meter from other people.	32(100)	0(0)	98(97)	3(3)
	-Taking antibiotics	1(3.1)	31(96.9)	3(3)	98(97)
	- Taking some medicinal herbs such as garlic …	2(6.2)	30(93.8)	13(12.9)	88(87.1)
	- The vaccine for this virus already exists	0(0)	32(100)	1(1)	100(99)

Source: Prepared by the authors from the study results

Regarding attitudes, the safety measures taken by the Moroccan government to contain the spread of the pandemic were considered very important by the majority of participants (76.69%), fair and insufficient by the other participants (22.5%). Indeed, 68.8% of cancer patients versus 42.6% of relatives considered these safety measures as a barrier to access certain oncology care (supplementary table 04).

**Supplementary table 04:** Participants' views on the safety measures taken by the Moroccan state in relation to the continuity of oncology care between May, 20 2020 and June, 5 2020

In your opinion, the security measures taken by the Moroccan state, can be a barrier for cancer patients to:	Cancer patients' group		Relatives group	
	YES	NO	YES	NO
	N (%)	N (%)	N (%)	N (%)
-Carrying out health check-ups and analyses in relation to cancer?	22(68.8)	10(31.2)	43(42.6)	58(57.4)
-Follow the periodic cancer treatment protocol (chemotherapy, radiotherapy…?	21(65.6)	11(34.4)	46(45.5)	55(54.5)
-Follow the post-therapy monitoring schedule with the oncologist?	13(40.6)	19(59.4)	53(52.5)	48(47.5)
- Consult the doctor for conditions other than COVID-19?	9(28.1)	23(71.9)	38(37.6)	63(62.4)

Source: Prepared by the authors from the study results

Results also showed that while more than half of relatives (52.5%) did not expect to be affected by the virus, 84.4% of cancer patients were very worried. They were concerned by the potential risk of infection and felt personally at risk (93.8%) of serious complications from COVID-19 (supplementary table 05). in addition, participants expressed their fear of unruly people's behaviors not respecting the barriers and protective measures during confinement (supplementary table 06).

**Supplementary table 05:** Population at risk for COVID-19, according to Moroccan participants between May, 20 2020 and June, 5 2020

If you think this pandemic is dangerous, for whom in particular?	Cancer patients' group		Relatives group	
	YES	NO	YES	NO
	N (%)	N (%)	N (%)	N (%)
Cancer patients	30(93.8)	2(6.2)	63(62.4)	38(37.6)
The elderly	20(62.5)	12(37.5)	71(70.3)	30(29.7)
Patients with chronic pathologies	23(71.9)	9(28.1)	83(82.2)	18(17.8)
Children	5(15.6)	27(84.4)	9(8.9)	92(91.1)

Source: Prepared by the authors from the study results

**Supplementary table 06:** Moroccan participants' attitudes toward unruly individuals during confinement between May, 20 2020 and June, 5 2020

What do you think about people who are unruly and who do not respect the barriers and protection measures against the pandemic?	Cancer patients' group		Relatives group	
	YES	NO	YES	NO
	N (%)	N (%)	N (%)	N (%)
- I am so afraid of their behaviour	31(96.9)	1(3.1)	63(62.4)	38(37.6)
- Their behavior will contribute to the spread of the virus, especially in the elderly and those with cancer and chronic diseases	32(100.0)	0(0.0)	89(88.1)	12(11.9)
- Their behaviour will be the cause of the appearance of clusters/foci	28(87.5)	4(12.5)	92(91.1)	9(8.9)
- Their behavior will be the cause of the extended period of confinement	28(87.5)	4(12.5)	89(88.1)	12(11.9)

Source: Prepared by the authors from the study results

As additional findings, ¾ of cancer patients versus only half of relatives confirmed the negative impact of containment and health emergency on their health status. The mean score was higher in cancer patients (6.5±2.3) than in relatives (4.3±2.6) (1min-10max). Among cancer patients, this score was significantly associated with perception of the COVID-19 danger and cancer pathology. Indeed, it was significantly higher in those who perceived COVID-19 as a dangerous infection than in those who perceived it as a common infection. While for relatives this score was significantly associated with age, it higher in younger relatives (<36 years).

The rates of correct response for the 16 questions on practices related to COVID-19 were ranging from 75% to 100% for cancer patients and 31.3% to 100% for relatives. The mean practice score was 46.1±3.2 for cancer patients and 40.8±5.4 for relatives (0min-48max). In addition, the global rate of response was 96.0% for cancer patients and 85% for relatives (mean score*100 /total score). All of cancer patients, versus 98% of relatives were able to score above 24, which represents a very high level of COVID-19 preventive practices.

Although the level of practices of the two groups was very satisfactory, we note that, 96.9% of cancer patients against 64.4% of relatives, greet their colleagues and friends always from afar. While, 96.9% of cancer patients against 80.2% of relatives still or frequently (16%) wore masks when leaving home throughout the period of confinement. Only a small minority of cancer patients (6.3%) reported wearing a mask to avoid police arrests against (14.9%) of relatives. 96.9% of cancer patients versus 76.2% of relatives absolutely respect social distancing and just 9.4% of cancer patients versus 28.7% of relatives sometimes resort to hand washing by water only. While a proportion of cancer patients (21.9%) and relatives (37.7%) categorically refuse or hesitate a lot about the administration of the vaccine if it exists.

The other results of the degree to which participants applied individual safety and prevention measures against COVID-19 are presented in Supplementary table 07.

**Supplementary table 07:** Evaluation of Moroccan participants' practices between May, 20 2020 and June, 5 2020

Practices: Degree of application of safety measures and individual prevention measures	Cancer patients' group				Relatives group			
	Always	Frequently	Rarely	Never	Always	Frequently	Rarely	Never
	N (%)	N (%)	N (%)	N (%)	N (%)	N (%)	N (%)	N (%)
- When I meet my friends and colleagues, I always greet them from afar.	31 (96.9)	1 (3.1)	0 (0)	0 (0)	65 (64.3)	9 (8.9)	4 (4)	23 (22.8)
- I stay at home and only go out in case of extreme emergency.	32 (100)	0 (0)	0 (0)	0 (0)	82 (81.2)	16(15.8)	2 (2)	1 (1)
- I wash my hands with soap and water regularly and for enough time.	30 (93.8)	2 (6.3)	0 (0)	0 (0)	74 (73.2)	22 (21.8)	5 (5)	0 (0)
- I use the hydro-alcoholic solution for hand disinfection without soap and water.	28 (87.5)	2 (6.3)	1 (3.1)	1 (3.1)	61 (60.4)	24 (23.8)	15 (14.8)	1 (1)
- I wash my hands with water only	0 (0)	1 (3.1)	2 (6.3)	29(90.6)	3 (3)	6 (5.9)	20 (19.8)	72(71.3)
- I wear a mask to protect myself and others from the risk of infection.	31 (96.9)	1 (3.1)	0 (0)	0 (0)	81 (80.2)	16 (15.8)	2 (2)	2 (2)
- I put on a mask to avoid police arrests.	2 (6.2)	0 (0)	0 (0)	30 (93.8)	4 (4)	3 (3)	8 (7.9)	86(85.1)
- I respect the safety distance of at least one meter with other people.	31 (96.9)	1 (3.1)	0 (0)	0 (0)	77 (76.2)	22 (21.8)	2 (2)	0 (0)
- If I have any of the symptoms associated with the disease, I will go into isolation and inform the health authorities.	30 (93.8)	2 (6.3)	0 (0)	0 (0)	80 (79.2)	15 (14.8)	3 (3)	3 (3)
- If I find that I have contacted someone who is infected with the virus, I agree to be isolated at home for a period of time until it is proven that I am free of the disease.	31 (96.9)	1 (3.1)	0 (0)	0 (0)	83 (82.1)	13 (12.9)	1 (1)	4 (4)
- If I find that I have contacted a person infected with the virus, I agree to be isolated in an isolation unit for a period of time until I am proven to be free of the disease.	32 (100)	0 (0)	0 (0)	0 (0)	80 (79.2)	16 (15.8)	3 (3)	2 (2)
- If there is a laboratory test available for the detection of the virus, I am willing to do it.	31 (96.9)	1 (3.1)	0 (0)	0 (0)	83 (82.1)	9 (8.9)	4 (4)	5 (5)
- If there is a vaccine against the virus, I am willing to do it.	22 (68.7)	3 (9.4)	6 (18.8)	1 (3.1)	48 (47.5)	15 (14.8)	13 (12.9)	25 (24.8)
- I follow daily all the news related to the spread of the virus in my country.	29 (90.6)	1 (3.1)	2 (6.3)	0 (0)	66 (65.3)	19 (18.8)	13 (12.9)	3 (3)
- If programs or lectures (or other) containing information about the disease are available, I will listen to them and follow the instructions mentioned in them	26 (81.3)	5 (15.6)	1 (3.1)	0 (0)	36 (35.6)	41 (40.6)	14 (13.9)	10 (9.9)
- If protective measures and equipment are available at an affordable price, I will buy them to protect myself.	29 (90.6)	2 (6.3)	1 (3.1)	0 (0)	78 (77.2)	20 (19.8)	0 (0)	3 (3)

Source: Prepared by the authors from the study results

It was also noted that cancer patients have better practices than relatives. In this sample, no association was demonstrated between the cancer patients' practices score and the other variables under study. While this score was associated among relatives with professional activity and perception of the COVID-19 contamination risk. Non-active relatives and those who perceive COVID-19 as a dangerous infection reported better practices than active individuals or those who perceive it as a common infection.

The associations between the selected variables and the knowledge score, the practice score, the number of outings outside the house per week and the impact of containment on the health of participants are shown in Table 02. Furthermore, no link was found between these scores and educational attainment or marital status.

**Table 02:** Knowledge score, practice score, number of outings/week and the impact score of confinement on the health of the two groups of Moroccan participants relative to sociodemographic data and perception of the danger of having covid-19 between May, 20 2020 and June, 5 2020

			Knowledge score				Practice score		Number of outings outside the house /week			Impact score of confinement on health		
	Variables		N (Mean±SD^d^)		p^e^	N (Mean±SD)		P	N	(Mean±SD)	P	N	(Mean ± SD)	P
	Groups	Cancer patients	32	22 ± 2.9	0.563	32	46.1 ± 3.2	0	32	1.5 ± 2.4	0	32	6.5 ± 2.3	0
		Families	101	21.5± 3.1	0.568	101	40.8 ± 5.4		101	2.7 ± 2.2		101	4.3 ± 2.6	
	
Cancer patients	Sex	Male	8	22.6 ± 2.0		8	46.5 ± 4.2	0.101	8	0.8 ± 0.9	0.708	8	7.3 ± 1.8	0.323
		Female	24	21.8 ± 3.1	24	46.0 ± 2.9	24		1.7 ± 2.7	24		6.3 ± 2.5		
	Age	<36 years old	6	20.2 ± 3.3	0.093	6	47.7 ± 8.2	0.121	6	2.8 ± 2.0		6	6.2 ± 2.8	
		≥ 36 years old	26	22.4 ± 2.7		26	45.8 ± 3.5		26	0.8 ± 1.4	0.018	26	6.6 ± 2.3	0.697
	Environment	Urban	29	21.9 ± 3.0	0.535	29	46.4 ± 2.7	0.316	29	1.3 ± 1.8	0.384	29	6.4 ±2.3	0.602
		Rural	3	23.0 ± 2.6		3	43.3 ± 6.4		3	0.3 ± 0.6		3	07 ±3.5	
	total household income	≤3000 dh	9	20.7 ± 3.3	0.144	9	45.4 ± 4.0	0.516	9	3.2 ± 3.6	0.029	9	6.0 ± 1.9	0.374
		>3000 dh	23	22.5 ± 2.6		23	46.4 ± 2.9		23	0.8 ± 1.3		23	6.7 ±2.5	
	Professional activity	Active	16	23.0 ± 2.7	0.036	16	46.5 ± 2.9	0.631	16	0.9 ± 1.4	0.429	16	6.3 ± 2.7	0.648
		Not active	16	21.0 ± 2.9		16	45.6 ± 3.5		16	1.5 ± 1.9		16	6.8 ± 1.9	
	Medical insurance	Yes	28	22.3 ± 2.8	0.177	28	46.5 ± 2.6	0.36	28	0.9 ± 1.4	0.057	28	6.6 ± 2.3	0.455
		No	4	20.3 ± 3.3		4	43.3 ± 5.9		4	3.3 ± 2.4		4	5.8 ± 2.4	
	Perception of covid-19 severity	None/low	2	19.0 ± 1.4		2	47.0 ± 1.4	0.931	2	1.5 ± 2.1		2	2.5 ± 2.1	
		Medium/high	30	22.2 ± 2.9	0.135	30	46.1 ± 3.3		30	1.2 ± 1.7	0.801	30	6.8 ± 2.1	0.041
Relatives	Sex	Male	33	21.2 ± 2.7	0.233	33	39.9 ± 4.4	0.069	33	3.6 ± 2.0	0.002	33	3.8 ± 2.5	0.177
		Female	68	21.7 ± 3.2		68	41.2 ± 5.8		68	2.2 ± 2.1		68	4.6 ± 2.7	
	Age	<36 years old 52		21.7 ± 2.8		52	41.5 ± 3.9	0.305	52	2.6 ± 2.3		52	4.9 ± 2.4	
		≥ 36 years old	49	21.3 ± 3.3	0.634	49	39.9 ± 6.6		49	2.8 ± 2.0	0.624	49	3.7 ± 2.7	0.01
	Environment	Urban	89	21.7 ± 3.1	0.017	89	40.7 ± 5.6	0.95	89	2.7 ± 2.1	0.459	89	4.3 ± 2.7	1
		Rural	12	20.0 ± 2,5		12	41.2 ± 4.1		12	2.3 ± 2.5		12	4.2 ± 2.4	
	total household income	≤3000 dh	12	21.0 ± 3.0	0.461	12	42.2 ± 4.5	0.398	12	1.9 ± 1.8	0.234	12	4.2 ± 2.1	0.897
		>3000 dh	88	21.6 ± 3.1		88	40.5 ± 5.5		88	2.8 ± 2.2		88	4.3 ± 2.7	
	Professional activity	Active	78	21.6 ± 3.3	0.289	78	40.1 ± 5.6	0.038	78	3.2 ± 2.2	0	78	4.4 ± 2.7	0.696
		Not active	23	21.4 ± 2.1		23	42.9 ± 3.8		23	1.1 ± 1.1		23	4.1 ± 2.5	
	Medical insurance	Yes	86	21.7 ± 3.0	0.032	86	40.7 ± 5.3	0.691	86	2.9 ± 2.1	0.009	86	4.3 ± 2.7	0.827
		No	15	20.3 ± 2.7		15	41.0 ± 6.2		15	1.4 ± 1.9		15	4.2 ± 2.0	
	Perception of covid-19 severity	None/low	20	20.9 ± 4.4	0.959	20	37.6 ± 6.4		20	2.9 ± 2.1		20	3.7 ± 3.0	0.169
		Medium/high	81	21.7 ± 2.6		81	41.5 ± 4.9	0.006	81	2.6 ± 2.2	0.64	81	4.5 ± 2.5	

d standard deviation

e p-value

Source: Prepared by the authors from the study results

During the period of containment, 65.6% of cancer patients attended hospital for treatment or cancer-related check-ups. 21.9% of them claimed to have found it difficult to access this care. 100% of these patients were able to comply with the diet prescribed by their doctors, while 50% of them confirmed the impossibility of practicing physical activity at home.

In the last seven days prior to the survey, the average number of outings outside the house per week was 1.5±2.4 for cancer patients compared to 2.7±2.2 for relatives. It should be noted that 50% of cancer patients versus 20.79% of relatives never left their homes. 37.5% of cancer patients versus 41.58% of relatives left home one to three times a week. 12.5% of cancer patients versus 37.62% of relatives left home more than four times a week. The reason for leaving home more than four times a week was mainly for an absolute necessity or for work. It should be noted that cancer patients leave home less frequently than relatives. The number of outings per week was significantly and positively associated with age and income. For relatives, it was associated with gender, medical insurance and employment. Indeed, active relatives, males and those with medical insurance, left their homes during containment, more frequently than non-active relatives, females and those with no medical insurance (table 02).

In parallel with the successful implementation of safety and personal protection measures, cancer patients reported having made significant changes in their relationships with other people 62.5% vs 9.9% for relatives and their daily habits 65.6% vs 17.8% for relatives as a result of the pandemic.

## Discussion

COVID-19 is a new emerging pandemic that is evolving very rapidly. It is a highly contagious disease. The risk of complications is clearly approved among the most vulnerable groups, including cancer and immunocompromised patients, as well as the elderly and people with chronic diseases 8,9.

In this uncertain pandemic context with the absence of a specific treatment or universal vaccine against all variants of SARS-CoV-2, Morocco has taken drastic measures to prevent community contamination. Preventive measures remain the only way to contain the pandemic and minimize its impact on the Moroccan health system and the health of the population vulnerable to the disease. For this reason, the assessment of KAPs of certain high-risk categories is necessary.

Results showed a female predominance in respondents and a fairly high level of education for relatives. The majority of the respondents were from urban areas and they had a medium socio-economic level.

A global rate of response on knowledge of 84.6% for cancer patients versus 82.7% for relatives, indicated that most respondents are familiar with COVID-19. These data corroborate with those from the KAPs studies of Egyptians 22, Chinese residents 18, general public in the United States and the United Kingdom 23, and a Bi-national survey in Africa 24. While other studies have shown low levels of knowledge among Malaysians 25, adults with chronic conditions in the United States 13 and Ethiopians 26. The knowledge score was associated only with the professional activity, the environment and medical insurance. Similar studies showed other significant relationships with gender, age, education, professional activity, family income, marital status, and ethnicity 18,22-24,26,27.

The vast majority of cancer patients (96.0%) and relatives (85%) had also a very high global rate of response about the practices against the COVID-19. These results are similar to those of studies conducted among Egyptians 22, Chinese residents 18, general public in the United States and the United Kingdom 23, and the bi-national survey in Africa 24. In contrast to the results from the Sudanese population 27. significant associations were demonstrated in this study between practice score and professional activity, age, gender, income, medical insurance, cancer and perception of the COVID-19 contamination risk.

Despite the very high level of practices for both groups, it was noted that some participants confirmed the use of water alone for hand washing, others wore face masks just to avoid police arrests, while others did not respect social distancing. However, WHO strongly recommends respect to effective hand washing with soap and water for at least 20 seconds, that the mask be worn systematically, and that social distancing be respected, pending an effective vaccine or treatment against covid-19 28.

The study also showed that the majority of cancer patients (84.4%) were very worried and concerned about the potential risk of COVID-19. They felt personally at risk of getting the disease and suffering from serious complications (93.8%). These results corroborate with those of the Milan study of young cancer patients 29, Egyptians 22, adults with chronic diseases in the United States 13 and Ethiopia 26.

Despite these concerns, a significant proportion of cancer patients (21.9%) and relatives (37.7%) were found to be categorically unwilling or very reluctant to receive the vaccine if it is available. Other studies reveal the same observation, such as the study in Egypt (11.5%) 22 and that of Israel 30. this finding may be related to conspiracy theory, the phenomenon of politicization of vaccines or the lack of confidence in the vaccines being carried out and merits further investigation 17,30,31.

## Conclusion

Up to this time, few studies have begun to evaluate KAPs in cancer patients and relatives, either at the national or international level. We believe that the present study is the first in Morocco to address this topic in this pandemic context. It has demonstrated the positive impact of the measures taken by the Moroccan government on participants' KAPs levels. The study also has some limitations. It is an online survey that may exclude other categories of the Moroccan population who do not have access to the Internet and social media and probably those with a very low level of education. In addition, the generalization of the study results is difficult, given the limited sample size. While the data collection tool used is a conceptual tool, adapted from the literature review, it has not been previously validated to assess KAPs on COVID-19.

In general, participants in this survey had good knowledge of COVID-19 and good safety measurement practices. This is a very favourable element in stemming the spread of the pandemic among at-risk populations. However, knowledge, attitudes and practice scores were associated primarily with age, gender, Professional activity, medical insurance area of residence, presence or absence of cancer, and perception of the risk of contracting the COVID-19.

The analysis of the characteristics of the KAPs of cancer patients and relatives regarding the COVID-19 has identified some associated factors. Other factors and aspects deserve to be explored through further studies. Knowing these factors is of great importance for decision-makers in Morocco. They enable them to inform decision making during the development and implementation of communication plans and to conduct effective outreach campaigns. They allow the identification of target groups to better contain the COVID-19 pandemic and all emerging infections.
